# Supplementary material for: Landslide topology uncovers failure movements
Source: Nat Commun. 2024 Mar 25;15:2633. doi: 10.1038/s41467-024-46741-7 (PMC10963756; doi:10.1038/s41467-024-46741-7)
Supplement: Supplementary file 1 — Supplementary Information [file 41467_2024_46741_MOESM1_ESM.pdf]

SI: Landslide topology uncovers failure movements

Kushanav Bhuyan[1,2*†], Kamal Rana[3,2,4*†], Joaquin Vicente Consunji Ferrer[4,5], Fabrice Cotton[2,6], Ugur Ozturk[2,4], Filippo Catani[1] and Nishant Malik[7]

[1]Machine Intelligence and Slope Stability Laboratory, Department of Geosciences, University of Padova, Padova, 35129, Veneto, Italy.
[2]Helmholtz Centre Potsdam – GFZ German Research Centre for Geosciences, Potsdam, 14473, Brandenburg, Germany.
[3]Chester F. Carlson Center for Imaging Science, Rochester Institute of Technology, Rochester, 14623, NY, USA.
[4]Institute of Environmental Science and Geography, University of Potsdam Potsdam, 14473, Brandenburg, Germany.
[5]Potsdam Institute for Climate Impact Research, Potsdam, 14473, Brandenburg, Germany.
[6]Institute of Geosciences, University of Potsdam, Potsdam, 14473, Brandenburg, Germany.
[7]School of Mathematics and Statistics, Rochester Institute of Technology, Rochester, 14623, NY, USA.

*Corresponding author(s). E-mail(s): kushanav.bhuyan@phd.unipd.it; kr7843@rit.edu;
Contributing authors: ferrer@uni-potsdam.de; fcotton@gfz-potsdam.de; oeztuerk@gfz-potsdam.de ; filippo.catani@unipd.it; nxmsma@rit.edu;
†These authors contributed equally.

Note S1. Introduction

This supplementary information (SI) accompanying the manuscript titled "Landslide Topology Uncovers Failure Movements" provides a comprehensive analysis of landslide topology and its significance in identifying landslide failure types based on their movement. The SI offers an analysis of topological features and their probability distributions, quantifying variations among different failure types and the relationship between landslide topology and physical processes. Additionally, it notes observations on the distinctions between topology and geometry in differentiating failure movements, includes ancillary sensitivity analyses conducted in scenarios with limited data, and verifies previously undocumented landslide inventories. It also details information on coupled landslide movements within complex landslide scenarios, discusses the method's impact on debris avalanches, and concludes with remarks on the method's technical limitations. Lastly, the SI presents measures to evaluate model performance, ensuring a robust assessment of the method's efficacy in their predictions.

Note S2. Behavior of different failure types

The inherent differences between failure types, notably their kinematic and mechanical behaviors, contribute prominent intricacies to the topography (see surface profiles in Figure 1 in the main manuscript). These intricacies are attributed to slope deformity, interior deformation, kinematic width of failures while propagating down-slope, main scarp deformation, run-out length represented by the debris/earth/soil transportation, and accumulated debris, and others captured by topology. The following are some of the most common failure types and their various behaviors.

The profile of rotational slides is marked by a conspicuous primary scarp and a distinctive back-tilted bench at the head, but little interior deformation (a schematic view can be seen in Figure 1 in the main manuscript). They are typically slowly moving a large portion of the weak rock mass. At the same time, kinematically rapid planar sliding is marked by the sliding of a rock mass on a planar rupture surface with little to no internal deformation, where the scarp might be separated from the stable rock at deep vertical tension cracks. Typically, they exhibit very compact shapes. Cohesion, c plays an important role in slides, as the degree of internal strength between the particles in a block of material determines the strength and stability along the slip plane. Translational landslides, like the ancient Seimareh slide in Iran's Zagros Mountains, are among the largest and most destructive on Earth.[12]

Flows are characterized by very rapid movements consisting of saturated granular material on moderate slopes, including liquefaction of materials (in the context of co-seismic triggers) or excess pore pressure (in the context of rainfall triggers) originating from the landslide source. When the internal friction angle, φ, is low (due to the mixture of solid and fluid particles), less external force is required to instantiate a failure because they are displaced

quite easily. Kinematically flow-ish movements are observed with channelized streams and a bulk deposit of debris at the talus (deposition zone), representing highly elliptical, elongated bodies.[7]

Usually limited in volume, falls (particularly rock falls) exhibit ballistic movements (high velocity, energy, and momentum) that are massively destructive. They detach from cliffs and move at high velocities, either by rolling, falling, or bouncing due to the influence of gravity. The run-out of a rock fall is often shorter and is more likely to travel along a straight path, whereas the run-out of debris flows is longer and can meander and spread out over a wider area.[3]

Complex failures are very hard to describe, as there is an amalgamation of different failure types occurring at the same time or subsequently, and they can therefore exhibit multiple characteristics of other failure types. For example, irregular debris slides evolving into a debris flow or any other combination of slides, flows, and falls eventually evolving into another movement style can be considered examples of complex failure.[6,9]

Such morphological and geometrical information for each distinct failure type is theorized to be captured in the topological space by the topological properties, which are then utilized in the machine-learning model to identify the failure types.

Note S3. Topological Features

Persistence diagrams capture the life-death information of structures like connected components, holes, and voids. The persistence diagram consists of a set of $\{(b_i, d_i)\}_{i=1}^{i=N}$ pairs corresponding to each structure type; here, i and N are the indexes of birth-death pairs and the total number of the birth-death pairs. Using the set of $\{(b_i, d_i)\}_{i=1}^{i=N}$ pairs, we can calculate various topological features such as persistence entropy, average lifetime, number of points, Betti curve-based measure, persistence landscape curve-based measure, Wasserstein amplitude, Bottleneck amplitude, Heat kernel-based measure, and landscape image-based measure.

Some of the above topological features can be explained using a lifetime vector that is calculated using a set of $\{(b_i, d_i)\}_{i=1}^{i=N}$ pairs. The lifetime vector $[l_i]_{i=1}^{i=N}$ is calculated as the difference between death and life of the (b_i, d_i) pair ($l_i = d_i - b_i$). The number of points, average lifetime, and persistence entropy are the length, average, and Shannon entropy of the lifetime vector. In comparison, topological features like Bottleneck and Wasserstein's amplitudes quantifying the magnitude of the lifetime vector are p-norm (p=2) and ∞-norm of the lifetime vector, respectively.

The Betti curve-based feature is a p-norm of a 1D discretized betti curve, which is a function ($B(\epsilon) : \mathbb{R} \to \mathbb{Z}$) mapping the persistence diagram to an integer-valued curve, and it counts the number of birth-death pairs at a given ϵ, satisfying the condition $b_i < \epsilon < d_i$.[8] Similarly, a persistence landscape curve-based feature is a p-norm of a 1D discretized persistence landscape curve

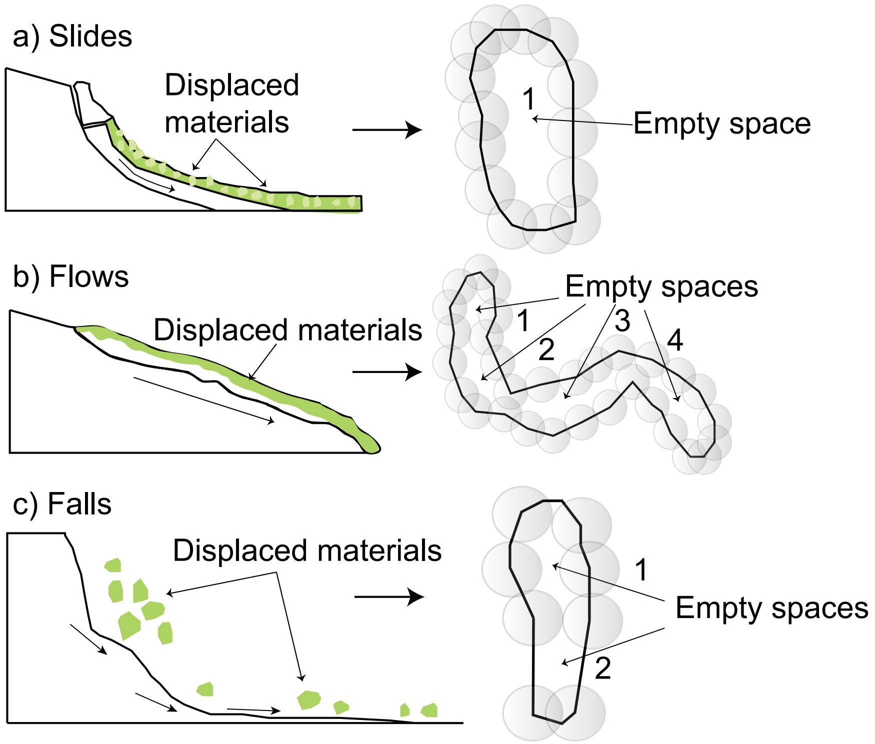

Figure S1: **Illustration of topological components on typical landslide movements.** The diagram shows a schematic outlook of how empty spaces are created in different landslide types (green color expresses the displaced material for each movement type). This illustration is shown using a simplified 2D transformation of a rather complex 3D topological phenomenon for ease of understanding. Sub-plots (a–c) refer to the possible configurations of empty spaces created in the typical polygons of each failure type. Slides tend to have the fewest empty spaces or holes due to their compact shapes, followed by falls. Flow-type failures tend to have multiple numbers of empty spaces due to the sinuous shapes they conjure as they follow the landscape's channelized topography.

defined as $\lambda(k, \epsilon) : \mathbb{R} \to \mathbb{R}_+$, where $\lambda(k, \epsilon) = k_{max}\{f_{b_i,d_i}(\epsilon)\}_{i=1}^{i=n}$, k_{max} is the k-th largest value of a set of functions defined by $f_{b_i,d_i}(\epsilon) = max\{0, min(\epsilon - b_i, d_i - \epsilon)\}$ for each (b_i, d_i) pair.[4]

The heat kernel-based feature is a p-norm (p=2) of the discretized 2D function obtained using the operation of the heat kernel on the persistence diagram. Heat kernel uses a gaussian kernel (σ) and a negative of the gaussian kernel (σ) for each (b_i, d_i) pair and mirror of (b_i, d_i) pair across the diagonal.[11] In contrast, the persistence image-based feature is a p-norm (p=2) of the discretized 2D function obtained using the operation of the weighted Gaussian

kernel on all $(b_i, d_i - b_i)$ pairs in the birth-persistence diagram.[1] The birth-persistence diagram consists of $(b_i, d_i - b_i)$ pairs where the x-axis shows the birth information, and the y-axis shows the lifetime of the (b_i, d_i) pair.

Note S4. Geometric versus Topological Features

Geometric properties define an object's shape and size, but topological properties explain the connections and topological interactions among its parts. Geometric properties such as area, perimeter, convexity (smallest convex shape that encompasses all the points of the landslide area), and ellipticity (semi-minor-axis: shortest radius extending from the ellipse's center to its edge, capturing the narrowest width of the landslide's elliptical representation) define the physical dimensions of a landslide, whereas topological properties such as the average lifetime of holes, Betti curve, and landscape curve describe the connections and interactions of the soil and rock masses, the width of kinematic propagation, and the depth of failure in a landslide. Geometric properties are, however, sensitive to any changes made to the original shapes of the geometry and, therefore, more susceptible to drastically changing the geometric property values. For example, any change to a landslide's boundary/body would inadvertently change each of the values of geometric properties like area, perimeter, convexity, etc., but the same cannot be said for topology, as it relies on the number of voids that are generated based on the overall shape of the landslide body. This is even more pronounced upon investigating the landslides in 3D. Since these geometric properties cannot be broadcasted to 3D, much information related to variational changes in the topography (attributed to elevation and slope) is lost. As TDA captures this 3D information and utilizes it when engineering topological features, intricate information on landslides such as depth of failure, deformation pattern, and the width of kinematic progression is well recorded.

Therefore, to assess and evaluate the differences between the classical and topological properties, we compare them in this section. This comparison was based on KDE plots that represent the PDFs of the samples for each failure type. We also plotted box plots to compare the median values and distribution of said values between the geometric and topological properties. As we see in Figure S2-b, the PDFs of the failure types are very similar to each other, specifically when looking at the ellipticity, semi-major axis, perimeter, and width. However, when comparing them to the topological properties Figure S2-a, we observe that the PDFs of the failure types are more dissimilar to each other under each property (e.g., the average lifetime of holes, bottleneck amplitude of holes, Wasserstein amplitude of holes). This can be the reason why the random forest models show promising results, as the PDFs are dissimilar enough to find evident differences between each failure type when using the topological properties/features. This effective ability to distinguish failure movements is also seen across 1) limited samples (the US Pacific Northwest and Wenchuan,

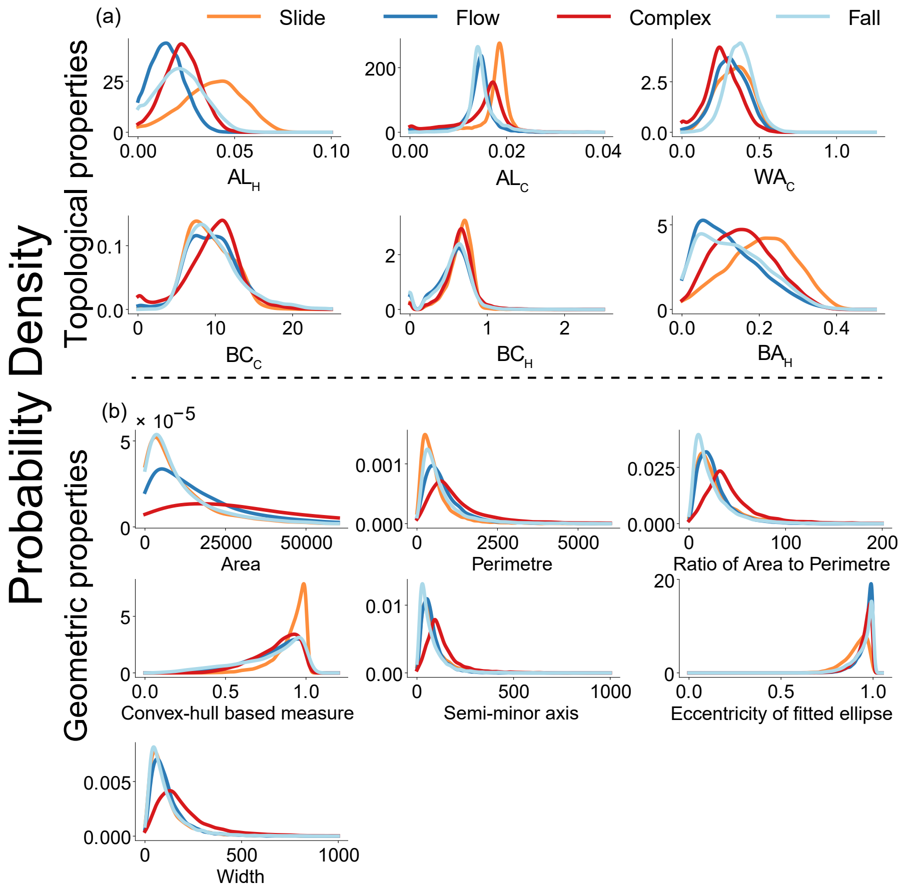

Figure S2: **Topological and geometrical feature statistics.** Probability distribution functions of the geometrical and the topological features for each failure type– slides (colored in orange), flows (colored in dark blue), complex (colored in red), and falls (colored in light blue)–in Italy. The y-axis shows the probability density values (calculated using kernel density estimation), and the x-axis shows the value of topological or geometrical attributes. The topological properties in plot-a) are: Average lifetime of holes ($\mathrm{AL_H}$), Average lifetime of connected components ($\mathrm{AL_C}$), Wasserstein amplitude of holes ($\mathrm{WA_H}$), Betti-curve based feature of connected components ($\mathrm{BC_C}$), Betti-curve based feature of holes ($\mathrm{BC_H}$), and Bottleneck amplitude of holes ($\mathrm{BA_H}$). The geometric features are: area (A), perimeter (P), the ratio of area to perimeter $\frac{A}{P}$, convex hull-based measure (C_h), minor(s_m), and width (W) of the minimum area bounding box fitted to the polygon.

China; Figure S3), 2) temporal prediction of debris flows and debris slides in Wenchuan, China, (Figures S4 and S5), and 3) coupled failure movements under complex landslide scenarios (Figure S6).

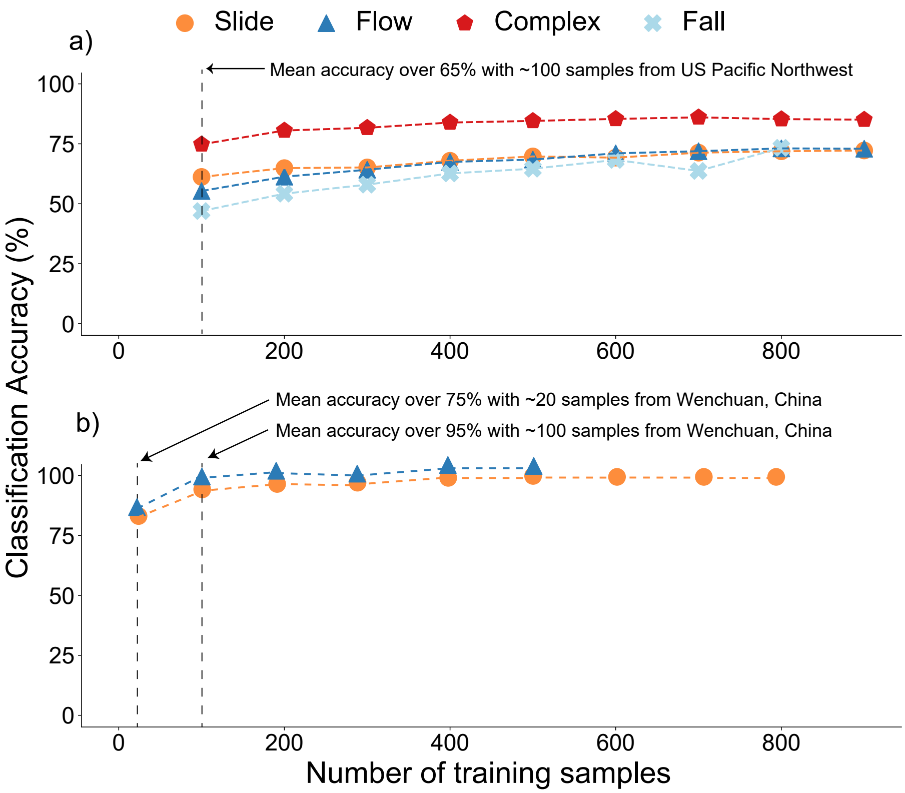

Figure S3: **Sensitivity analysis on limited training samples for effective predictions on the US Pacific Northwest and Wenchuan, China.** The figure shows the classification accuracy (in%) corresponding to each failure type (slides: colored in orange, flows: colored in dark blue, complex: colored in red, and falls: colored in light blue) with the number of training samples for a) the US Pacific Northwest and b) Wenchuan, China. The x-axis shows the number of training samples from each class used to train the model, and the y-axis shows the classification accuracy (in%) corresponding to each class. At 100 samples, the mean classification accuracy reaches over 65% in the US Pacific Northwest and 95% in Wenchuan, China. Even at 20 samples, the performance reaches 75%. The regions of Denmark and Turkey were not tested due to a lack of samples within the classes of fall-type (62 samples) and complex-type (92 samples) within these inventories, respectively.

Note S5. Verification of landslides in 2005 and 2007 Wenchuan, China multi-temporal inventory

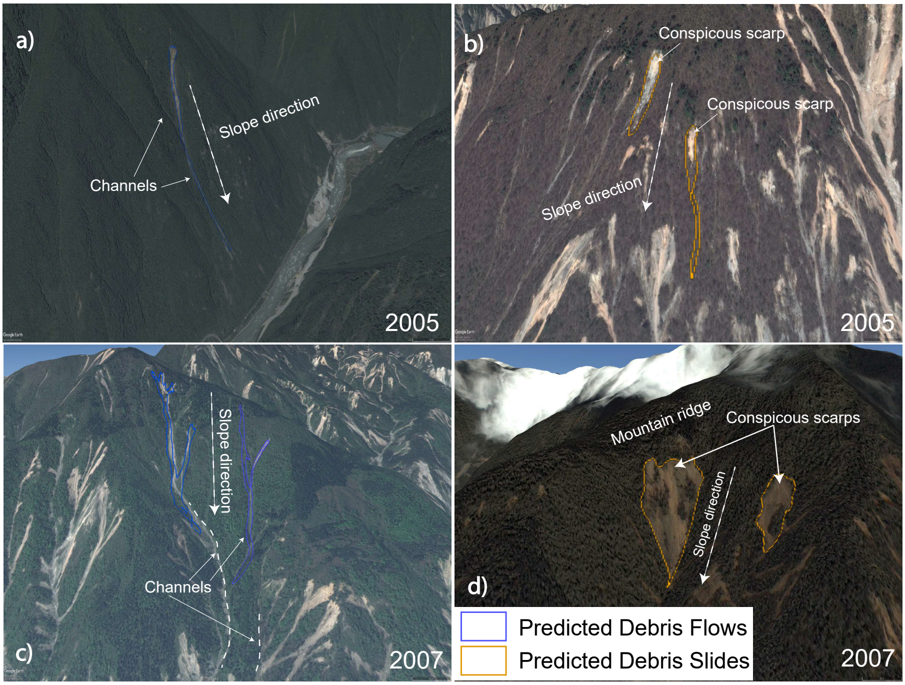

Figure S4: **Verification of 2005 and 2007 landslides using Google Earth imagery.** Panels (a) and (b) display model predictions for the 2005 inventory, while panels (c) and (d) pertain to the 2007 inventory. The analysis of certain features, like conspicuous or prominent scarps, channels, mountain ridges, and travel distances, aids in understanding failure movements. The plots illustrate predictions for both debris slides (colored in orange) and flows (colored in dark blue), using these features to corroborate and verify the two types of movements.

To verify landslide movement types from Google Earth images, we focus on specific geomorphological features that are characteristic of different types of landslides (debris slides and debris flows in the case of Wenchuan, China). The approach is as follows:

- **Examination of debris flow characteristics**:
  To identify debris flows, we searched for channelized flow patterns.[6] This phenomenon is crucial in differentiating debris flows from other landslide types. Our attention was also directed towards the recognition of channels

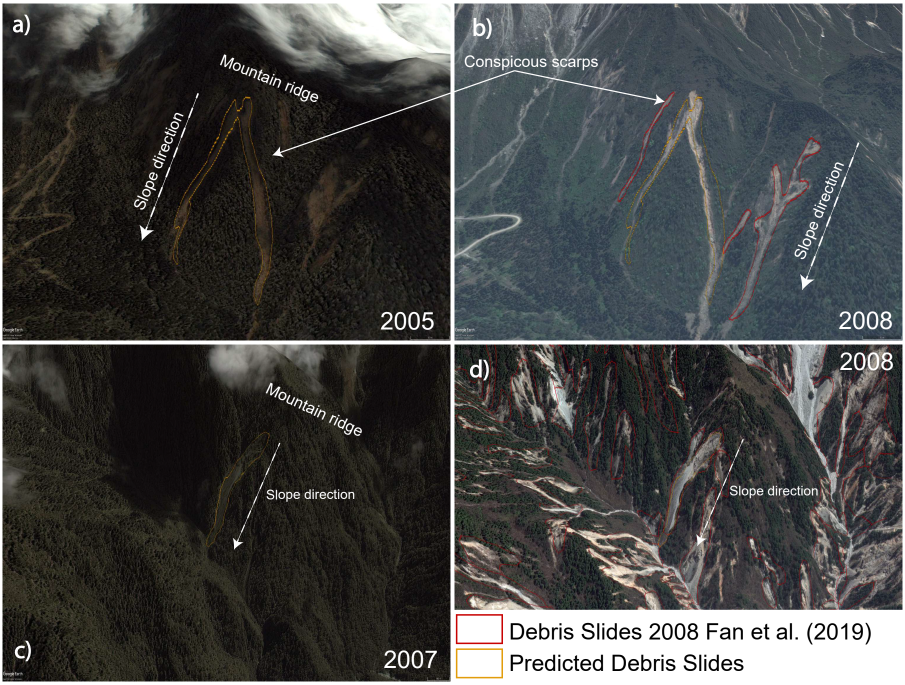

Figure S5: **Verification of 2005 and 2007 landslides using archived temporal Google Earth imagery.** Panels (a) and (c) show model predictions for landslides (debris slides: colored in orange and debris flows: colored in dark blue) in 2005 and 2007, while panels (b) and (d) show landslides identified by Fan et al. (2019) after the 2008 Wenchuan earthquake. Archived Google Earth images are used to track landscape changes on slopes affected by landslides post 2005 and 2007.

along the flow paths (Figure S4-a and c), indicative of channelized debris transportation, a hallmark of debris flow activity.[9] Furthermore, we also assessed the sinuosity and travel distances of these flows, as we suspect the hillslope channelized debris flow to harbor longer travel distances than debris slides.[13]

- **Examination of debris slide characteristics**:
  We looked for abrupt, steep cliff-like features in the terrain where prominent scarps (see Figure S4-b and d) are often indicate debris slides and/or shallow slides[14]). Additionally, many debris slides were observed on the ridge of the mountains, necessitating further scrutiny of these landslides in such topographic conditions. Another key aspect of our analysis involved assessing the sinuosity and travel distance. Typically, debris slides exhibit less sinuous paths with shorter travel distances than debris flows.[13] Therefore, we looked for settings where such topographic conditions were met to qualify and confirm the movements as debris slides.

- **Archive imagery to track landscape changes**:
  We also employed the timeline feature of Google Earth images to conduct a more nuanced and comprehensive understanding of the individual landslides, their evolution, and what is occurring nearby in the topography to better understand the typology. We looked at the initiations, pathways, accumulations, terrain changes, and material deposition along the channels and hillslopes to better assess the movement type. By systematically examining historical imagery, we were able to verify, for example, the movement and accumulation of debris, providing more insights into verifying the predictions made by the model more effectively (see Figure S5).

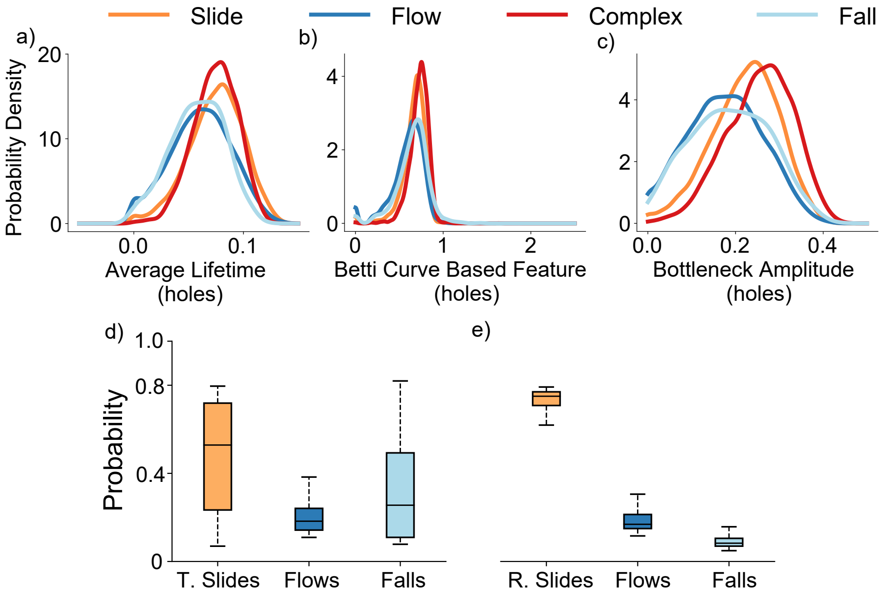

Figure S6: **Exploration of coupled failure movements within complex landslides with topological features.** Sub-plots (a-c) display the probability distribution functions for three key topological properties for the US Pacific Northwest: Average Lifetime of holes, Betti curve-based features of holes, and Bottleneck amplitude of holes. These distributions reveal a striking similarity between sliding-type and complex landslide failures. Sub-plots (d-e) show the probability of complex landslides belonging to each of the failure types class (slide: colored in orange, flow: colored in dark blue, and fall: colored in light blue) as predicted by the model. Box plot (d) shows the complex landslide samples that occur from "Translational rock slides followed by rock falls" as documented in the Statewide Landslide Information Database for Oregon (SLIDO). Model predictions indicate sliding failure to be the predominant type of failure, which are most likely translational slides according to SLIDO. Similarly, box plot (e) shows the complex landslide samples that occur from "Rotational slides followed by flows" as recorded in SLIDO. Model predictions indicate slide type to be the predominant type of failure, which most likely ruptures rotationally. Beige bars illustrate sliding mechanisms while bars with darker and lighter shades of blue illustrate flows and fall types, respectively. Note the number of annotated complex failures with behavioral definitions by SLIDO in box plot (a) constitutes 198 samples and box plot (b) constitutes 230 samples.

Note S6. Topology and outlook towards debris avalanches

Debris avalanches, categorized within the realm of flow-type movements, often occur in topographical settings similar to those conducive to debris slides.[9] Consequently, the morphological and material characteristics of debris slides and debris avalanches may exhibit notable similarities. This observation is corroborated by the fact that debris slides are frequently categorized as a preliminary stage in the development of debris avalanches (and also debris flows), serving as a mechanism of initiation.[9] In the context of debris avalanches, we anticipate that their movement will impart a topographic signature that, while generally akin to that of debris slides, remains distinct. Specifically, debris avalanches are characterized by forming a debris fan (conical shape) at the deposition part, a feature amiss in debris slides. These avalanche deposits have distinct imprints on the landscape, thereby influencing the topology of the landslide polygonal shape, which Topological Data Analysis (TDA) can effectively discern. Although the initial stages of both debris slides and avalanches may exhibit similar morphologies, this variation in the deposition area of the landslide can be leveraged by our model to distinguish between debris slides and debris avalanches. In consensus, as long as there are distinct variations in shapes amongst the different movement types, our model can distinguish them, even between debris slides and avalanches.

A caveat in the approach can stem when the model's performance is conditioned on the preciseness of the avalanches mapped in the inventory. These inventories are primarily sourced/generated from expert observations (both in the field and remotely) and institutional reports. This reliance may inadvertently introduce a bias, particularly in differentiating between landslide types with subtle distinctions in their topographical and material characteristics. A pertinent example could be the challenge of separating debris avalanches from debris slides.

Broadly, debris slides are grouped as an initial component of debris flows or debris avalanches, in which they function as an initiation mechanism.[9] This feature truly indicates that landslides are quite complex by nature, where a failure event could manifest more than one mode of movement (for example, a landslide transitioning from initial soil cracking to a debris avalanche and finally into a surging sediment-laden channel, collectively termed as 'debris flow'). Our model, therefore, tends to classify landslides based on their most dominant or final observable state, potentially simplifying the multifaceted dynamics of landslide processes. For instance, a debris slide evolving into a debris avalanche might predominantly be identified as the latter by our model since that would be the last imprint on the topography.

To address this limitation, a more nuanced model could be developed, focusing on initial indicators such as the scarp area, which may provide insights into the primary failure mechanism, thereby enhancing the model's classification

ability.[2] Alternatively, we can adapt existing data to consider the evolution stages (capturing and separating the moment when debris slides converge to an avalanche) but this would take more effort in re-curating the data to avoid misclassifications.

Note S7. Technical limitations of the method

Certain limitations can arise pertaining to the shape delineation of landslide bodies. Manual mapping efforts provide the most accurate representation of landslides when mapped from field or remote observations when compared to automated mapping. In the past years, despite automated tools gaining traction due to their reliability in rapid mapping and assessment,[5] their use in generating complete inventories (either event-based or historical) is still limited. These limitations arise from issues like amalgamations[10] where more than one landslide body is mapped together as a single entity due to similar spectral responses of the landslide pixels in satellite images. Inadvertently, it affects the assessment of reliable landslide statistics such as travel distance, propagation area, and volume estimates. For our model to operate properly, the amalgamation issue needs to be addressed as an intermediate step following mapping by automated tools. An avenue in this endeavor would be to explore the sensitivity between manual and automated inventories via TDA.

Furthermore, the method's effectiveness relies on the quality and geographical location of the training and testing data used in the model. Manual annotations of failure types can lead to bias since various mappers will have different perspectives (mapping on aerial or satellite imagery versus geomorphological field mapping can display distinct perceptions) when annotating the landslides and their failure types. Also, due to the ambiguous nature of complex-type failures, they could include slides and flows simultaneously, which can impact the overall performance of the model. The method's reliance on a DEM for converting 2D polygons into 3D shapes also presents potential challenges. DEM quality in the training and testing regions can bias the results, particularly for smaller landslides, as coarser DEM resolutions may struggle to capture the profiles of these smaller-scale events.

Note S8. Other measures to evaluate model performance

In order to evaluate the performance of the method, we also calculated the confusion matrix and other accuracy metrics like the True Positive Rate, True Negative Rate, False Positive Rate, False Negative Rate, and the F1-score.

The True positive rate (also known as Sensitivity, Recall) (equation 1) and true negative rate (also known as Specificity) (equation 2) are performance metrics that are used to assess a model's accuracy in accurately detecting positive and negative instances. The number of genuinely negative instances identified as positive by the model is known as false positives (FP) (equation

3). The number of cases that are genuinely positive but are categorized as negative by the model is known as false negatives (FN) (equation 4).

$$TPR\ (= Recall) = \frac{True\ Positives}{True\ Positives + False\ Negatives} \tag{1}$$

$$TNR = \frac{True\ Negatives}{True\ Negatives + False\ Positives} \tag{2}$$

$$FPR = \frac{False\ Positives}{True\ Negatives + False\ Positives} \tag{3}$$

$$FNR = \frac{False\ Negatives}{True\ Positives + False\ Negatives} \tag{4}$$

The F1-score (equation 6) is the harmonic mean of precision (equation 5) and recall (equation 1), and it is used to balance the precision-to-recall trade-off. Precision is the number of correct positive predictions produced by the model out of all correct positive predictions made by the model, and recall is the number of correct positive predictions made out of all correct positive occurrences.

$$Precision = \frac{True\ Positives}{True\ Positives + False\ Positives} \tag{5}$$

$$F1\text{-}score = 2 \cdot \frac{Precision \cdot Recall}{Precision + Recall} \tag{6}$$

In Figure S7, we see the confusion matrix and the respective scores of the TPR, TNR, FPR, FNR, and F1-score of both Italy and the US Pacific Northwest.

Predicted Class

Actual Class	Slide	Flow	Complex	Fall
Slide	**98.60**	0.00	1.18	0.22
Flow	0.0	**94.77**	1.70	3.53
Complex	0.66	1.99	**96.39**	0.96
Fall	0.15	2.80	0.96	**96.11**

Metrics	Slide	Flow	Complex	Fall
TPR	98.50	94.69	96.27	96.00
TNR	99.69	98.38	98.67	98.40
FPR	0.30	1.61	1.32	1.59
FNR	1.49	5.30	3.73	3.97
F1-Score	**98.79**	**94.90**	**96.16**	**95.64**

Figure S7: **Accuracy statistics for the Italian data.** The figure shows the confusion matrix and the associated accuracy metrics of the random forest model for the data of Italy.

References

[1] H. Adams, T. Emerson, M. Kirby, R. Neville, C. Peterson, P. Shipman, S. Chepushtanova, E. Hanson, F. Motta, and L. Ziegelmeier. Persistence images: A stable vector representation of persistent homology. *Journal of Machine Learning Research*, 18, 2017.

[2] I. Baroň, J. Jelének, J. Klimeš, J.-J. Dong, R. Melichar, M. Šutjak, Y. Chen, C.-M. Yang, E.-L. Zhang, J. Méndez, C.-H. Tseng, F. Hartvich, J. Blahůt, T.-T. Nguyn, L. Kociánová, F. Bárta, V. Dušek, and P. Kycl. Source area morphometry and high depletion rate of landslides may indicate their coseismic origin. *Engineering Geology*, 330:107424, 2024.

[3] F. Bourrier, L. Dorren, and O. Hungr. The use of ballistic trajectory and granular flow models in predicting rockfall propagation. *Earth Surface Processes and Landforms*, 38(4):435–440, 2013.

[4] P. Bubenik and P. Dłotko. A persistence landscapes toolbox for topological statistics. *Journal of Symbolic Computation*, 78:91–114, 2017.

[5] N. Casagli, E. Intrieri, V. Tofani, G. Gigli, and F. Raspini. Landslide detection, monitoring and prediction with remote-sensing techniques. *Nature Reviews Earth & Environment*, 4(1):51–64, 2023.

[6] D. Cruden and D. Varnes. Landslide, investigation and mitigation. *Transportation Research Board Special Report 247*, 1996.

[7] S. Evans, M. Bovis, and J. Hutchinson. Landslides of the flow type. *Environmental & Engineering Geoscience*, 7(3):221–238, 2001.

[8] A. Garin and G. Tauzin. A topological "reading" lesson: Classification of mnist using tda. In *2019 18th IEEE International Conference On Machine Learning And Applications (ICMLA)*, pages 1551–1556. IEEE, 2019.

[9] O. Hungr, S. Leroueil, and L. Picarelli. The Varnes classification of landslide types, an update. *Landslides*, 11(2):167–194, 2014.

[10] O. Marc and N. Hovius. Amalgamation in landslide maps: effects and automatic detection. *Natural Hazards and Earth System Science*, 15(4):723–733, 2015.

[11] J. Reininghaus, S. Huber, U. Bauer, and R. Kwitt. A stable multi-scale kernel for topological machine learning. In *Proceedings of the IEEE conference on computer vision and pattern recognition*, pages 4741–4748, 2015.

[12] N. J. Roberts and S. G. Evans. The gigantic Seymareh (Saidmarreh) rock avalanche, Zagros Fold–Thrust Belt, Iran. *Journal of the Geological Society*, 170(4):685–700, 2013.

[13] B. Strîmbu. Modeling the travel distances of debris flows and debris slides: quantifying hillside morphology. *Annals of Forest Research*, pages 119–134, 2011.

[14] D. J. Varnes. Slope movement types and processes. *TRB Special Report*, 176:11–33, 1978.
